# Ultrasound-Induced Changes in Physicochemical, Microstructural, and Antioxidative Properties of Whey-Protein-Concentrate-Encapsulated 3,3′-Diindolylmethane Nanoparticles

**DOI:** 10.3390/antiox14030273

**Published:** 2025-02-26

**Authors:** Abbas Khan, Cuina Wang, Adam Killpartrick, Mingruo Guo

**Affiliations:** 1 Department of Nutrition and Health Promotion, University of Home Economics Lahore, Lahore 54700, Pakistan; abbaskhan9916@mails.jlu.edu.cn; 2 College of Food Science and Engineering, Jilin University, Changchun 130062, China; wangcuina@jlu.edu.cn; 3 College of Agriculture and Life Sciences, The University of Vermont, Burlington, VT 05405, USA; adam.killpartrick@uvm.edu

**Keywords:** 3,3′-diindolylmethane (DIM), whey protein, nanoparticles, encapsulation, ultrasound

## Abstract

This study determined the impact of ultrasound duration on the encapsulation of 3,3′-diindolylmethane (DIM) using whey protein concentrate (WPC) nanoparticles. Whey-protein-concentrate-based DIM nanoparticles were prepared and treated with different ultrasound times (0–20 min) with 30% amplitude at 4 °C. The results showed that ultrasound treatment significantly decreased the mean particle size (from 265 nm to 218 nm) and the Polydispersity Index (PDI) value (from 0.49 to 0.43) as well as zeta potential values were notably increased. The encapsulation efficiency (EE%) increased with increasing sonication time (0–20 min) from 76% to 88%, respectively. The ultrasound treatment had a significant effect on the apparent viscosity, and a decrease in the viscosity as a function of shear rate was observed with increasing sonication time. The transmission electronic microscopy (TEM) micrographs demonstrated that all of the formulations treated with different sonication times had a smooth and uniform spherical shape and ultrasound treatment led to the reduction of particle size, especially after 20 min of ultrasound. The thermal stability of the WPC–DIM nanoparticles was enhanced with increasing sonication time by increasing peak denaturation temperature and enthalpy. The Fourier transform infrared spectroscopy (FT-IR) spectra analysis revealed that ultrasound treatment had a remarkable effect on the secondary structure of WPC–DIM nanoparticles; electrostatic interactions and hydrogen bonds between DIM and whey protein were strengthened. Moreover, the length of ultrasound treatment exhibited a significant effect on the DPPH (2,2-diphenyl-2-picrylhydrazyl) scavenging activity (from 56% to 62%) and ABTS(2,2′-azinobis(2 ethylbenzothiazoline-6-sulfonate) scavenging activity (from 47% to 68%). In conclusion, the ultrasound treatment successfully improved the physicochemical, microstructural, and antioxidative properties of WPC–DIM nanoparticles; therefore, it is considered an effective method to develop whey-protein-concentrate-based DIM nanoparticles for medical and nutritional applications.

## 1. Introduction

3,3′-Diindolylmethane (DIM) is a naturally occurring lipophilic compound found in the *Brassica* family, most notably kale, broccoli, and cauliflower. DIM has demonstrated beneficial effects on human health due to its strong therapeutic and medicinal properties, such as anticancer [1], antioxidant [2], anti-inflammatory [3], and anti-bacterial [4]. Most importantly, its antioxidant effect has shown an inhibitory effect on inflammation, tumor growth, oncogenesis, and progression. These properties suggest the use of DIM as a chemopreventive or chemotherapeutic agent in cancer patients with improved Quality of Life (QoL) [2,5]. Many studies reported the anticancer effect of DIM in vitro and in vivo experimental models and clinical trials [6,7]. It is confirmed in the literature that DIM, when consumed even at high levels, exhibits a low level of toxicity, suggesting its use as a nutraceutical [5]. However, its clinical advancements and broad-spectrum applications are hampered in the food and pharmaceutical industries because it is degraded easily when exposed to light and heat [8,9], and is unstable at a low pH and susceptible to oxidative degradation [10]. Moreover, its absorption is limited in the gastrointestinal tract due to its high lipophilicity and low solubility, which reduces its overall oral bioavailability [11]. To overcome these limitations, there is a need to develop an enhanced bio-available orally active pharmaceutical dosage of DIM to prevent various diseases.

Nano-encapsulation techniques have gained more attention as a delivery mechanism for the better protection of bioactive components mainly due to their various properties, for example, enhanced bioavailability, better permeability, and solubility [12]. In order to enhance the oral bioavailability and stability of bioactive compounds, numerous delivery systems have been used [13]. Whey proteins are considered to be a suitable wall material for the nano-encapsulation of bioactive compounds [14,15]. Whey is a by-product and dietary supplement of the dairy industry, consisting of two major globular proteins, such as β-lactoglobulin and a-lactalbumin [16]. In the past decade, whey protein nanoparticles have been employed as a coating agent to protect many sensitive bioactive compounds (curcumin, theophylline, and totarol) from heat, light, and oxygen, as well as to improve bioavailability [17,18,19]. Previously, whey protein nanoparticles have been developed by using numerous methods, including heat-induced polymerization by heating whey protein above 80 °C for a prolonged time [10,15], and spray drying [20] to encapsulate bioactive agents. Many studies are conducted involving the novel technologies, such as the use of ultrasound or other means to microencapsulate bioactive compounds with the improved physicochemical properties of whey proteins [21,22]. The effect of ultrasound on the physicochemical and anti-microbial properties of whey protein–totarol nanoparticles was investigated. The results revealed that the physicochemical, rheological, and anti-microbial properties of whey-protein-based nanoparticles were improved with the increase in ultrasound treatment (10~30 min) [19]. Ultrasound is an innovative, green, novel, non-thermal processing technology, commonly used in the dairy and related industries to modify the protein’s physicochemical and functional properties [19]. Ultrasound treatment boosts the likelihood of contact between the bioactive compounds, which can enhance the stability of the protein–polysaccharide complex because of its special cavitational effects [23]. Nanoencapsulation with innovative technology is the best choice to protect the efficiency of DIM during harsh environmental conditions and enhance oral bioavailability [13,24]. Ultrasound treatment with various sonication times and frequencies was utilized to enhance the physicochemical, rheological, functional, and micro-structure properties of bioactive compounds encapsulated in various delivery systems [19,23,25]. Based on the previously reported ultrasonic modification and impact mechanism, we hypothesized that ultrasound treatment may lead to modifications in the physicochemical, micro-structural, and antioxidative properties of WPC–DIM nanoparticles, with enhanced binding affinity and encapsulation efficiency (EE). Therefore, this study determined the impact of ultrasound duration on the encapsulation of 3,3′-diindolylmethane (DIM) using whey protein concentrate (WPC) nanoparticles.

## 2. Materials and Methods

### 2.1. Materials and Chemicals

3,3′-Diindolylmethane (DIM) was obtained from Luotian Xinpusheng Pharmaceutical Co., Ltd. (Huanggang, China). De-ionized Milli-Q pure water was used from a Milli-Q water filtration system (Millipore Corp., Bedford, MA, USA). Whey protein concentrate (WPC) was provided by Hilmar (Turlock, CA, USA).

### 2.2. Preparation of Nanoparticles

Freshly prepared whey protein concentrate (WPC) solution (8%, *w*/*v*) was prepared by dispersing whey protein concentrate powder in de-ionized Milli-Q pure water. At ambient temperature, the solution was stirred at (1500 rpm) for 6 h and was kept overnight at 4 °C to achieve full dissolution. The pH of the solution with 2 M NaOH was adjusted to pH 7.0 and equilibrated at room temperature. Heating protocol: WPC solution was heated for 15 min at 80 °C, while stirring at 1800 rpm in a water bath. The sample was kept in cold water for 30 min and then stored at ambient temperature for further treatment.

3,3′-Diindolylmethane (DIM) stock solution was prepared by dissolving DIM in pure ethanol (15 mg/mL) as described by Khan et al. [10]. The solution was then gently stirred at 1500 rpm overnight to achieve a clear solution [10].

For the preparation of DIM-encapsulated WPC nanoparticles, the measured amount of DIM was slowly dropped into WPC solution while stirring to achieve a ratio of 1:10 (*v*/*v*). The resultant solution was stirred overnight at 25 °C using a magnetic stirrer at 600 rpm. The mixture was then stored in glass bottles for further studies. The sodium azide (0.022%, *w*/*w*) was then mixed to inhibit the growth of microorganisms.

### 2.3. Ultrasound Treatment of WPC–DIM Nanoparticles

A 50 mL aliquot of the WPC–DIM nanoparticles for each treatment was poured into a jacketed beaker (300 mL) of the ultrasound with circulating cooled water. The samples were treated with different ultrasound times (0, 5, 10, 15, and 20 min) using an ultrasonic processor (VCX800, vibra cell, Sonics, Newtown, CT, USA) with a 13 mm high-grade titanium alloy ultrasound probe dipped in the samples (20 kHz; 10 s: 5 s work/rest cycles; 30% amplitude; 4 °C). A sample without ultrasound treatment was also prepared as a control. After ultrasound treatment, ethanol was removed by a rotary evaporator (EYELA N-1100, Tokyo, Japan) at 100 rpm and 37 °C [15]. Fresh nanoparticles were subjected to particle size, polydispersity index (PDI), zeta potential, and rheological measurement and transmission electronic microscopy (TEM).

### 2.4. Freeze Drying

The freshly prepared nanoparticles, WPC only and WPC–DIM nanoparticles with and without ultrasound treatment, were frozen at −80 °C, and then freeze dried using a freeze dryer for 24 h at −55 °C, 0.033 MPA (Alpha 1–2, Marin Christ Inc., Osterode, Germany). The sample powders obtained were packed in airtight bags and stored at a suitable temperature until further analysis.

### 2.5. Nanoparticles Characterization

#### 2.5.1. Particle Size, Polydispersity Index (PDI), and Zeta Potential

The particle size and PDI of the freshly prepared nanoparticles with and without ultrasound treatment were analyzed by Zetasizer (Nano-ZS, Malvern Instruments, Worcestershire, UK). The samples were diluted to (0.01%) using de-ionized Milli-Q pure water and filtered through (0.02 um). Then, 1 mL aliquot was dripped into the disposable zeta cell and equilibrated for 3 min before analyses at 25 °C with a 173° scattering angle. All of the samples were assessed three times and the particle size was reported as mean particle diameter (Z-average). The nanoparticles’ polydispersity index (PDI) was taken concurrently with the particle size measurement.

The zeta potential of the fresh nanoparticles treated with different ultrasound treatments (0, 5, 10, 15, and 20 min) was measured by dynamic light-scattering techniques using a Zetasizer (Nano-ZS, Malvern Instruments, Worcestershire, UK). The samples were diluted at a ratio of 1:1000 with de-ionized Milli-Q pure water and stirred for 30 s. Then, 1 mL of the aliquot was taken and placed in a clear disposable zeta cell and equilibrated for 3 min before analysis. The samples’ measurements were taken in triplicate.

#### 2.5.2. Encapsulation Efficiency (EE%)

The encapsulation efficiency (EE%) of the DIM in whey protein concentrate nanoparticles with different ultrasound treatment times was evaluated by the previous method with some modifications [9]. The free DIM (non-encapsulated) in the whey protein concentrate nanoparticles was determined as follows: twenty milligrams of the freeze-dried sample (prepared according to the method explained in 2.4. Freeze drying) was flushed three times with 5 mL ethyl acetate and was filtrated using filter paper (No.1 Whatman). The filtrate was then measured at 281 nm by a UV–visible spectrophotometer (UV-2550, Shimadzu, Tokyo, Japan) using an appropriate standard curve (*r*^2^ = 9.966). The DIM was extracted according to the previous method [15]. The washed sample was mixed with DMSO/methanol (1:1), and vortexed for 5 min followed by centrifugation at 8000× *g* for 10 min. The DIM was extracted from the supernatant and was analyzed using a UV–visible spectrophotometer (UV-2550, Shimadzu). The encapsulation efficiency was calculated using the following formula:

$$\mathrm{EE}\%=\frac{\mathrm{Total}\;\mathrm{DIM}\;\mathrm{amount}-\mathrm{Free}\;\mathrm{DIM}\;\mathrm{amount}}{\mathrm{Total}\;\mathrm{DIM}\;\mathrm{amount}}\times 100$$

### 2.6. Rheological Determination

Rheological properties (apparent viscosity) were determined using a hybrid rheometer (HR-1, TA Instruments, New Castle, DE, USA) fitted with a cooling system (Thermo Cube, Plymouth, IN, USA). The system was equipped with a 60 mm geometrical plate (1 mm thickness). All of the sample flow ramps were recorded in the shear rate range of 0.1 s^−1^ to 1000 s^−1^ at 25 °C. For each sample measurement, 10 mL of the sample was placed on the plate and thermal equilibration was allowed for 5 min before analysis. To avoid bubbles, care was taken in the sample.

### 2.7. Color and pH Measurement

The colors of WPC–DIM nanoparticles with and without ultrasound were measured using a CM 2300d Konica Minolta colorimeter (Osaka, Japan) according to CIE system (L*, a*, and b*). In this system, there is an L*-value (where a higher positive value represents lightness), a*-value (higher positive value represents redness), and b*-value (higher positive value represents yellowness). Before measuring the colors of the samples, the colorimeter was calibrated with black and white standards. Then, 10 mL of sample was poured onto a petri dish and measured against a black background in dark conditions for 3 repetitions. The color values were noted and analyzed.

The pHs of samples with and without ultrasound treatment were determined at room temperature. Each sample was put in a beaker and pH probe (Jenoy 3330, Vernon Hills, IL, USA) was immersed into the solution; readings were taken three times.

### 2.8. Transmission Electron Microscopy (TEM)

Effect of ultrasound treatment on the microstructure of WPC–DIM nanoparticles was determined by an H-7650 Transmission Electron Microscope (Hitachi High-Technologies, Tokyo, Japan) at 100 kV acceleration voltage. Briefly, nanoparticles were diluted at a ratio of 1:1000 with de-ionized Milli-Q pure water and stirred for 1 min. Then, a small amount (10 µL) of the sample was placed on carbon film copper grid and stained with 2% (*w*/*v*) uranyl accetate before imaging.

### 2.9. Differential Scanning Calorimetry (DSC)

The freeze-dried nanoparticles with various sonication times (0–20 min) were investigated for thermal properties using a Q2000 (DSC; TA Instruments) according to our previous method [10]. Nanoparticles were weighed (5–7 mg) in hermetically sealed aluminum pans (Tzero Pan, TA Instruments, New Castle, DE, USA) along with a blank pan (used as a reference) and were placed in the DSC equipment. The heating protocols were set from 20 to 250 °C (heating rate 10 °C/min) in an inert nitrogen gas atmosphere. Universal Analysis Software 3.9A (TA Instruments, New Castle, DE, USA) was used to determine the onset temperature (T_on_), peak denaturation temperature (T_d_), denaturation temperature range (ΔT_d_), and denaturation enthalpy (ΔH_d_).

### 2.10. Fourier Transform Infrared Spectroscopy (FT-IR)

FT-IR of the freeze-dried nanoparticles with and without ultrasound treatment was performed using FT-IR spectrophotometer (IR-Prestige 21, Shimadzu, Kyoto, Japan). Briefly, 2 mg samples were taken and were mixed with dried KBr (200 mg); the mixture was ground and pressed into slices. The spectra were acquired between 500–4500 cm^−1^ wavenumbers.

### 2.11. Antioxidant Activity

The effects of ultrasound treatment with various sonication times on antioxidant capacity of the WPC–DIM nanoparticles were investigated with 2 methods: DPPH (2,2-diphenyl-2-picrylhydrazyl) radical-scavenging activity and ABTS 2,2′-azinobis(2 ethylbenzothiazoline-6-sulfonate).

Samples of the WPC–DIM nanoparticles with different sonication times were diluted to 1 µL/mL in pure ethanol. DPPH stock solution (0.2 mM) was also prepared in pure ethanol. The sample and DPPH solution were mixed vigorously at 1:1 ratio and held in the dark at room temperature prior to analysis for 30 min. The absorbance of each solution was determined by a UV–visible spectrophotometer (UV-2550, Shimadzu) at 517 nm. 

For the ABTS evaluations, the nanoparticles with different sonication times were diluted to 1 µL/mL in pure ethanol. ABTS solution (7 mM) and potassium persulphate solution (2.45 mM) were developed in absolute methanol and vigorously mixed at a 1:1 ratio, and were kept in the dark for 16 h at −4 °C. Then, the solution was diluted with methanol until its absorbance reached 0.7 ± 0.02 to generate the ABTS^+^ assay solution. The sample was mixed at 1:10 ratio with ABTS solution and kept for 30 min in dark conditions at room temperature before analyses. The absorbance of ABTS/sample, blank, and ABTS were determined by UV–visible spectrophotometer (UV-2550, Shimadzu) at 734 nm.

$$\mathrm{AA}\;\%=\frac{1-\left(A_{s}-A_{b}\right)}{A_{d}}\times 100$$

where A_s_ denotes absorbance of DPPH/ABTS with samples, A_b_ represents absorbance of blank, and A_d_ is absorbance of DPPH/ABTS solution.

### 2.12. Statistical Analyses

All of the experiments were conducted three times and data were shown as mean ± SD. The data were subject to one-way ANOVA (analysis of variance) with a post hoc Tukey’s test using SPSS 20.0 (SPSS Inc., Chicago, IL, USA) at a significance level of (*p* < 0.05).

## 3. Results and Discussion

### 3.1. Effect of Ultrasound Treatment on the Particle Size, Polydispersity Index (PDI), and Zeta Potential of WPC–DIM Nanoparticles

Particle size is considered an important parameter determining stable nanoparticles in food and medical applications. The effect of ultrasound treatment using the ultrasonic probe with various times on the particle size and polydispersity index (PDI) of WPC–DIM nanoparticles was investigated. The nanoparticles were ultrasonicated at 30% amplitude for 0, 5, 10, 15, and 20 min. Table 1 summarizes the effect of sonication time on the particle size and PDI of WPC–DIM nanoparticles. The particle size of the native whey protein concentrate was around 230.50 nm, which was significantly increased (*p* < 0.05) to 265.96 nm (no ultrasound treatment) upon encapsulation of DIM [8]. This increase in the particle size occurred mainly because of the deposition of DIM in whey protein concentrate nanoparticles by the increased hydrophobic interaction between hydrogen bonding [10,26]. On other hand, after ultrasound treatment of WPC–DIM nanoparticles, a considerably significant reduction (*p* < 0.05) in the particle size of the WPC–DIM nanoparticles could be seen; for example, after ultrasound treatment for 5 min, the particle size of the untreated nanoparticle significantly dropped (*p* < 0.05) from 265.96 to 255.86 nm. This decrease in particle size could be attributed to the disruption of non-covalent bonds of whey proteins caused by the changes in hydrophobic and electrostatic interactions by high shear forces from ultrasonic cavitations [22,25]. The results were supported by the past literature, which revealed that ultrasound treatment could significantly decrease the average particle size of whey-protein-based nanoparticles [19]. Further, when the nanoparticles were treated with different ultrasound times (5, 10, and 15 min), a decrease in particle size with increasing sonication time could be seen, as shown in Table 1. However, ultrasound treatment for 20 min had the most significant (*p* < 0.05) influence on the particle size of the sample. The particle size appears to have been reduced by the cavitational forces produced by the probe; as additionally, the micro streaming and turbulence of the ultrasound broke the aggregates and masses, resulting in the smaller particle size of the nanoparticles [27]. Overall, a significant effect of ultrasound treatment (0–20 min) was recorded on the particle size of WPC–DIM nanoparticles. Many studies have reported on and proposed the use of the ultrasound technique for particle size reduction in nanoparticles with the increase in ultrasound treatment duration [19,28].

Polydispersity index (PDI) values indicate dimensionless measurement of the particle size distribution. The PDI value higher than 0.7 refers to a very high size distribution. The PDI values of all of the formulations are illustrated in Table 1. The PDI value of the WPC only was 0.41 ± 0.01 and was significantly increased (*p* < 0.05) to 0.49 ± 0.02 because of the entrapment of bioactive DIM in the whey protein concentrate nanoparticles. However, after ultrasound treatment for 5, 10, 15, and 20 min, the PDI value was significantly decreased (*p* < 0.05) to 0.48, 0.46, 0.43, and 0.43, respectively. This decrease in the PDI value suggests that ultrasound treatment broke down the large aggregates and brought more uniformity/a decrease in the particle size due to the homogenization effect [29]. The same results, i.e. that ultrasound treatment could result in a decrease in PDI values, were previously reported, when a compound was encapsulated in protein nanoparticles and was treated with different ultrasound frequencies [25].

Zeta potential indicates the surface charge characteristic of the samples, an important indicator to assess the stability of the colloidal system assessing their aggregation and dispersion. The greater the zeta potential, the greater the trend of dispersed particle is; hence, particles will repel each other, which will maintain a stable colloidal system. A zeta potential value above ± 30 mV in the colloidal system is considered enough for electrostatic repulsion to prevent coalescence and maintain a stable system [30]. The zeta potential values of the untreated and treated samples are given in Table 1. As seen, all of the samples had negative zeta potential values. The native whey protein concentrate zeta potential value was around −21 mV. After the formation of WPC–DIM nanoparticles (0 min ultrasound treatment), the zeta potential values significantly changed (*p* < 0.05) from −21.93 mV to −27.96 mV [8]. After ultrasound treatment for 5 min, the zeta potential values of WPC–DIM nanoparticles were significantly increased from −27.96 mV to −30 mV; however, ultrasonication for 10 and 15 min did not significantly impact the zeta potential of the nanoparticles, followed by a greater increase when the sample was treated for 20 min (−32 mV) (*p* < 0.05) (Table 1). Generally, these high-magnitude, negatively charged zeta potential values of the ultrasound treated samples suggest a desirable stable solution. It is clear from the results that more negatively charged amino acids are abundant in WPC–DIM nanoparticles [19]; hence, they showed that ultrasound treatment influenced the molecular interactions of WPC–DIM nanoparticles.

### 3.2. Effect of Ultrasound Treatment on the Encapsulation Efficiency (EE%) of WPC–DIM Nanoparticles

The effect of ultrasound treatment on the encapsulation efficiency (EE%) of DIM using whey protein concentrate nanoparticles is shown in Table 1. The results reported that ultrasound treatment with different sonication times enhanced the encapsulation efficiency (EE%) significantly (*p* < 0.05). The EE of DIM in whey protein concentrate nanoparticles without ultrasound was 76.4%, and was significantly improved to 77.3%, 79%, 81.5%, and 88.5% after ultrasound treatment for 5, 10, 15, and 20 min, respectively. These results suggest that ultrasound treatment remarkably improved the EE% of DIM compared to the untreated nanoparticles. Ultrasound treatment changes the molecular structure of whey protein concentrate and could increase the interaction between DIM and whey protein concentrate [22]. Also, sonication greatly improved the cavitation yield, collapse rate, and number of bubbles, which is considered to play an important role in enhancing the EE [23]. In addition, compact and homogeneous composite nanoparticles with a lower particle size were produced by strengthening the hydrogen bonding and electrostatic interactions under ultrasonic treatment, which may improve EE, which was confirmed by the TEM and particle size results.

### 3.3. Effect of Ultrasound Treatment on Rheological Properties of WPC–DIM Nanoparticles

**Figure 1:**
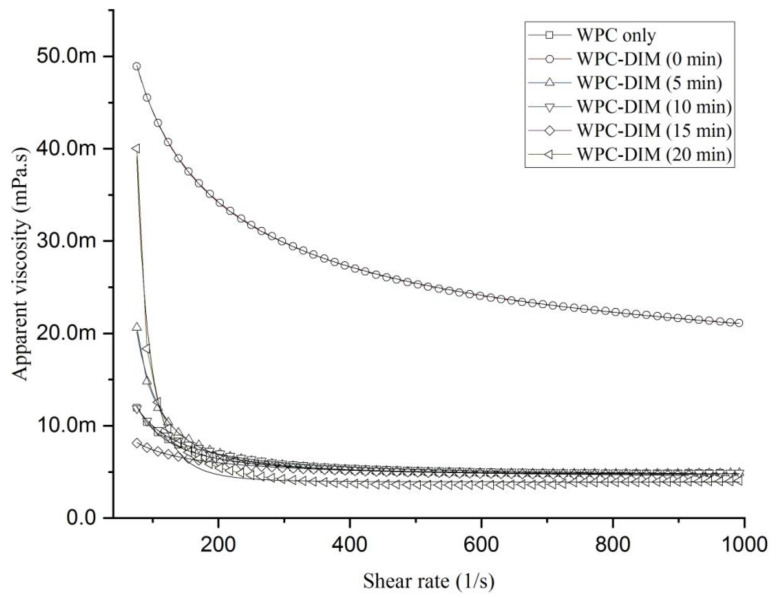
Effect of ultrasound treatment on rheological properties of WPC–DIM nanoparticles.

The shear forces produced by the ultrasonic cavitations may affect the rheological properties of the whey protein solutions by changing the degree of protein molecule aggregation inside the colloidal system [31]. Nanoparticles’ fluidity is mainly described by apparent viscosity [10]. Figure 1 shows the effect of ultrasound treatment with different sonication times (0–20 min) on the apparent viscosity (*η*_app_) of the WPC–DIM nanoparticles as a function of shear rate (γ). The viscosity of native whey protein concentrate was increased after encapsulation of DIM in the WPC nanoparticles. However, a significant decrease with increasing shear rate was observed with ultrasound treatment followed by an infinite-shear viscosity (*η*_∞_) at a high shear rate region. These results suggest the shear thinning and pseudo-plastic flow behavior of the samples, which remained pseudo-plastic after ultrasound treatments. The same behavior was reported previously [22]. All of the WPC–DIM nanoparticles treated with and without ultrasound conformed to Sisko’s model on the basis of strong correlation shown in Table 2, and showed shear-thinning behavior (*n* < 1) [22,32].

*η*_app_ = *η*_∞_ + *k*_ᴑ_ γ^*n*−1^

where *η*_∞_ is infinite shear rate viscosity, *k*_ᴑ_ value is the consistency index, and γ denotes the shear rate (s^−1^), while *n* is the flow index.

After ultrasound treatment, a significant decline in the infinite-shear viscosity (*η*_∞_) of the WPC–DIM nanoparticles, especially after 20 min of ultrasound treatment, was noted (*p* < 0.05). Our results revealed that the decrease in apparent viscosity was positively correlated with the change in infinite-shear viscosity (*η*_∞_), indicating that ultrasound treatments of various times significantly reduced the apparent viscosity of the samples (*p* < 0.05). The reduction in apparent viscosity may be due to the ultrasonic cavitation effect of ultrasound treatment. Sonication increases the surface hydrophobicity and reduces the protein aggregates, which could decrease the particle size, and this might be responsible for the rheological changes in nanoparticles and emulsions [27]. Also, the flow behavior was correlated with changes in particle size; previous findings reported that a reduction in the particle size of whey proteins could decrease the viscosity [27]. Moreover, a significant increase consistency index (*k*_ᴑ_) was recorded after ultrasound treatment (*p* < 0.05), followed by a slight decrease after 15 and 20 min of sonication. These results were in line with previous research [19,22]. The results suggest that ultrasound treatment decreased the viscosity of the WPC–DIM nanoparticles, which recommends its use in any low-viscosity foods.

### 3.4. Effect of Ultrasound Treatment on Color and pH of WPC–DIM Nanoparticles

Color is the most important factor that influences the consumer/client acceptability of a product. Phytochemicals often undergo some chemical changes such as isomerization reactions with ultrasound treatment due to the existence of free radicals produced by sonication treatment [33]. The effect of ultrasound treatment on color coordinates (CIE L*a*b*) of the WPC–DIM nanoparticles was assessed (Table 3). The results revealed that no significant effect of sonication with different times (5, 10, 15, and 20 min) on the color indicator L*-value of WPC–DIM nanoparticles was seen as compared to the control sample (0 min of ultrasound) (*p* < 0.05) (Table 3). Furthermore, no significant change (*p* < 0.05) was caused by ultrasonication for different times (0–10 min) to the a*-value and b*-value color parameters of the WPC–DIM nanoparticles, except for the ultrasound treatment of 20 min, where a significant change was noted for the b*-value. These findings suggest no effect on the color parameters of WPC–DIM nanoparticles by ultrasound treatment due to the appropriate selection of sonication duration, amplitude, and temperature. Moreover, DIM was successfully entrapped in whey protein concentrate nanoparticles and was not directly exposed. Our results were consistent with previous results, which reported that insignificant change was observed after the ultrasound treatment of milk protein. Ultrasound treatment has many advantages, such as enhanced foaming, physicochemical, and rheological properties; however, the inappropriate selection of time and frequency can denature proteins [34].

The pHs of all of the formulations were adjusted to 7.0 before ultrasound treatment. The effects of ultrasound treatment on the pH changes of WPC–DIM nanoparticles are illustrated in Table 3. Ultrasound treatment significantly increased the pH of WPC–DIM nanoparticles with increasing sonication time (*p* < 0.05). The pH of the WPC–DIM nanoparticles treated for 20 min increased by about 0.13 units from 6.95 to 7.08 under ultrasound as compared to the control sample. This increase might be due to the temperature and pressure in the near region of ultrasound-produced bubbles, which can induce changes in the protein structure and produce the free radicals that react with the protein side chain and can change or decrease the protein acidic group [27].

### 3.5. Effect of Ultrasound Treatment on Microstructure of WPC–DIM Nanoparticles

**Figure 2:**
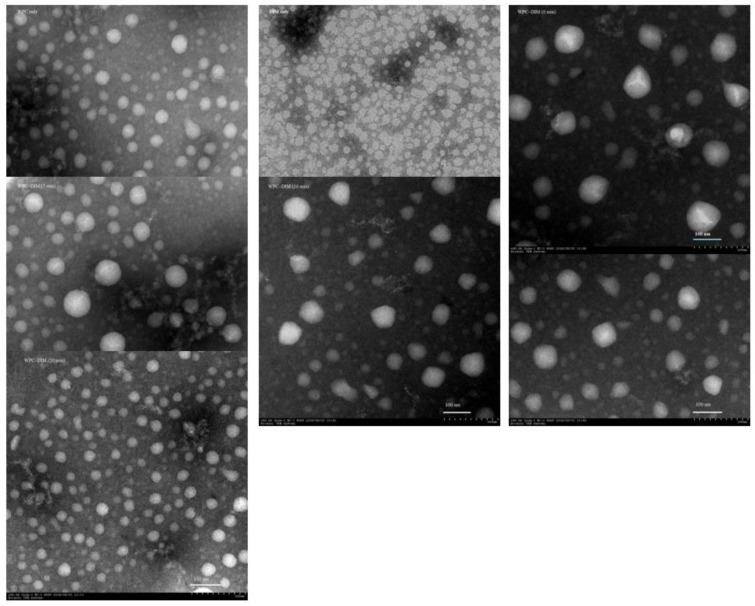
Effect of ultrasound treatment on microstructure properties of WPC–DIM nanoparticles.

The micrograph of the nanoparticles treated with various sonication times was examined using TEM (Figure 2). The native whey protein concentrate (WPC) exhibited a typical smooth and spherical shape, while the native DIM exhibited a rough and irregular shape, which is consistent with our previous findings (Figure 2) [8]. After adding DIM to whey protein nanoparticles, the nanoparticles with and without ultrasound treatment exhibited a spherical and smooth shape with a cloudy matrix of protein around the DIM. Furthermore, the findings indicate that the particle size of the WPC–DIM nanoparticles was greater than in comparison with the native whey protein concentrate, which could be attributed to the interaction of DIM and whey protein molecules [12]. The morphology of WPC–DIM nanoparticles changed notably after ultrasonication with different sonication times. As illustrated in Figure 2, the ultrasound-treated WPC–DIM nanoparticles had a spherical and smoother surface, having a smaller particle size as compared to the untreated sample, which was confirmed by the data obtained in the DLS study (Table 1). Recent research revealed that ultrasound treatment could reduce the particles size of the nanoparticles, loosen the protein structure, and result in the disclosure of more cleavage bonds [25,35]. Along with this, WPC–DIM nanoparticles with various sonication times showed a large surface and even a hollow structure, which is attributed to ultrasonication-induced changes (cavitations) [23]. The results show that ultrasound treatment strengthened the molecular interaction between DIM and whey protein concentrate by changing the spatial structure of the whey protein concentrate and widening the protein surface region, which could have improved the contact area between the whey protein concentrate and DIM [26].

### 3.6. Effect of Ultrasound Treatment on Differential Scanning Calorimetry (DSC) of WPC–DIM Nanoparticles

**Figure 3:**
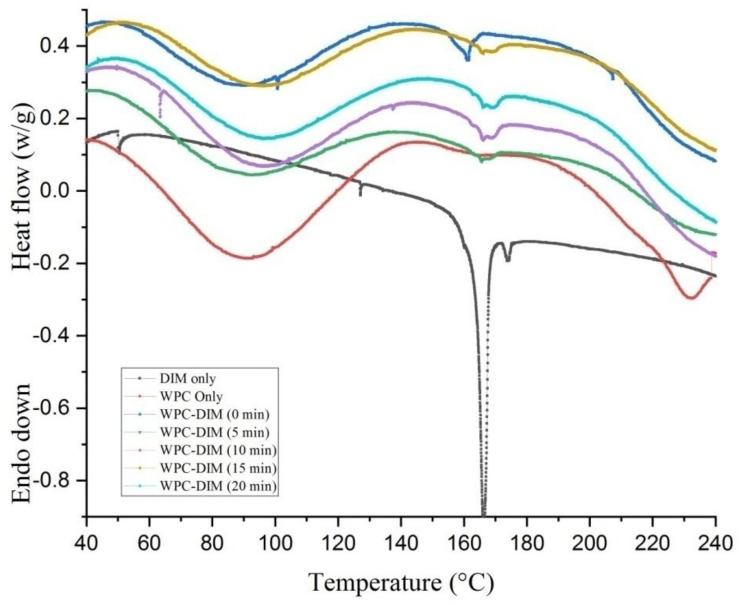
Effect of ultrasound treatment on differential scanning calorimetry (DSC) of WPC–DIM nanoparticles.

The thermal properties of native DIM, WPC only, and ultrasound-treated WPC–DIM nanoparticles with various sonication times (0–20 min) are shown in Figure 3. The onset temperature (T_on_), denaturation peak temperature (T_d_), denaturation temperature range (ΔT_d_), and denaturation enthalpy (ΔH_d_) were analyzed using a software and are presented in Table 4. The native DIM had a sharp endothermic peak around 170 °C and 175 °C, which suggests the melting temperature of DIM; a similar observation was previously reported [3]. Similarly, the native whey protein concentrate showed a broad endothermic peak at around 87 °C, which corresponds to heat-induced changes in β-lactoglobulin (the major component of whey protein concentrate) [10]. In addition, the denaturation peak temperature (T_d_) (90 °C) and denaturation enthalpy (ΔH_d_) (179 m W/g) were found to be almost similar to native whey protein concentrate. The thermal stability of the whey protein suggests it would be appropriate for nanoencapsulation of thermo-sensitive substances. Additionally, after sonication of WPC–DIM nanoparticles with various sonication times, the nanoparticles had much greater denaturation peak temperature (T_d_) and denaturation temperature range (ΔT_d_) as compared to the untreated sample (Table 4). After ultrasound treatment for 5, 10, 15, and 20 min, the T_d_s of the WPC–DIM nanoparticles were 90.57, 93.9, 94.96, and 96 °C, respectively, which were enhanced compared to the untreated sample (90.46 °C). In the same way, the ultrasound treatment shifted the denaturation temperature range (ΔT_d_) in comparison to the untreated sample (Table 4). These ultrasound-induced changes could be caused by the enhanced electrostatic and hydrogen bonding interactions between DIM and whey protein concentrate, which made the structure of WPC–DIM nanoparticles more compact. The ultrasonic treatment also enhanced thermal stability and formed new structures, and more energy would be required to break the macromolecular structures [23]. Moreover, ultrasound treatment can increase the melting temperature due to molecular aggregation by intermolecular hydrogen bonding interactions between hydrophilic head groups [25].

### 3.7. Effect of Ultrasound Treatment on Fourier Transform Infrared (FT-IR) Spectra of WPC–DIM Nanoparticles

**Figure 4:**
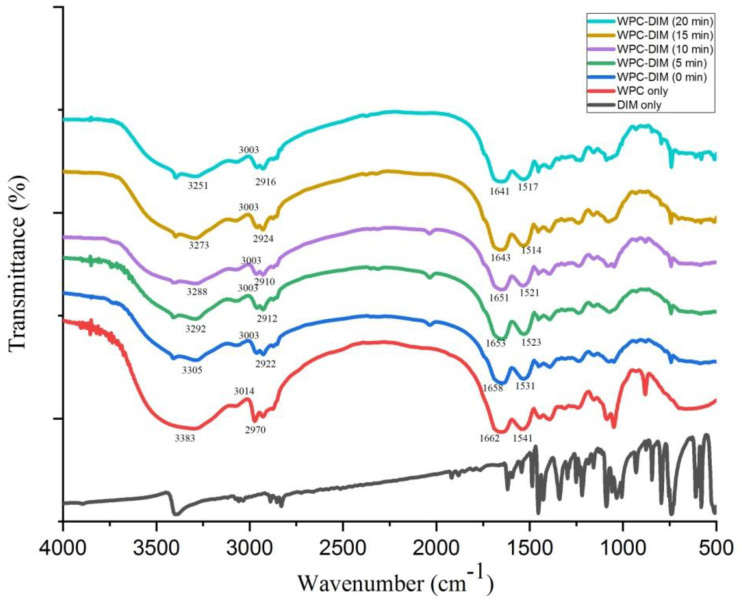
Effect of ultrasound treatment on Fourier transform infrared (FT-IR) spectra of WPC–DIM nanoparticles.

Fourier transform infrared (FT-IR) spectroscopy was applied to obtain the information about the intermolecular interactions inside WPC–DIM nanoparticles. The FTIR spectra of WPC only, native DIM, and WPC–DIM nanoparticles with various sonication times were recorded (Figure 4). The native whey protein has a prominent and broad absorption at 3383 cm^−1^ caused by the O–H stretching vibration. Also, the peak at 2970 cm^−1^ in native whey protein concentrate may be related to the C–H stretching vibrations, and the strong absorption peaks at 1662 cm^−1^ and 1541 cm^−1^ are related to the C=O stretching vibration and N–H bend. Similar results of whey protein concentrate FTIR spectra were previously observed [8]. After encapsulation of DIM in whey protein concentrate nanoparticles, the WPC–DIM nanoparticles without ultrasound showed interesting characteristic functional groups: the strong peak at 3305 cm^−1^ which relates to N–H stretching was shifted to 3292 cm^−1^, 3288 cm^−1^, 3273 cm^−1^, and 3251 cm^−1^ after ultrasound treatment for 5, 10, 15, and 20 min, respectively. Previous results confirmed that encapsulation using the ultrasound method can change hydrogen bonds from 3355 cm^−1^ to 3322 cm^−1^~3315 cm^−1^ [25]. The infrared spectrum of the WPC-only sample has changed from 3014 cm^−1^ to 3008 cm^−1^ (C–H stretching vibrations) in comparison to the WPC–DIM nanoparticles (without ultrasound), whereas no significant effect of the ultrasound treatment with various sonication times on C–H stretching vibrations was observed. The results were consistent with previous findings on the effect of ultrasound on the oil and water emulsion of whey protein [36]. The electrostatic interactions and hydrogen bonding of DIM and whey protein concentrate in the WPC–DIM nanoparticles was confirmed by the presence of an amide I band (1640–1690 cm^−1^, C=O stretch) and amide II band (1510–1560 cm^−1^, N-H bend) [37]. These findings were in line with our previous results; similar results were obtained by encapsulated DIM in polymerized whey protein [10]. After the sonication of nanoparticles with various sonication times, changes in the amide I and amide II wavelengths were observed and peaks were remarkably changed from 1658 cm^−1^ to 1653–1641 cm^−1^ and 1531 cm^−1^ to1523–1517 cm^−1^, respectively. In addition to this, the intensity of absorption in the amide I and amide II bands were increased (Figure 4), indicating the binding between DIM and whey protein concentrate [38]. Hence, these characteristic peaks suggest that more stable and strong hydrogen bonding and electrostatic interactions were obtained between DIM and whey protein concentrate after ultrasound treatment [25]. The amide I and amide II bonds were actively involved in hydrogen bonding between different elements contributing to the secondary structure of proteins. Many studies revealed that ultrasonication can change the secondary structure of proteins, such as α-helix, β-sheet and β-turn [36]. The electrostatic interactions and stronger hydrogen bonding recorded in the WPC–DIM nanoparticles after ultrasound treatment with various sonication times could be attributed to the change in the secondary structures of protein.

### 3.8. Effect of Ultrasound Treatment on Antioxidant Activity of WPC–DIM Nanoparticles

**Figure 5:**
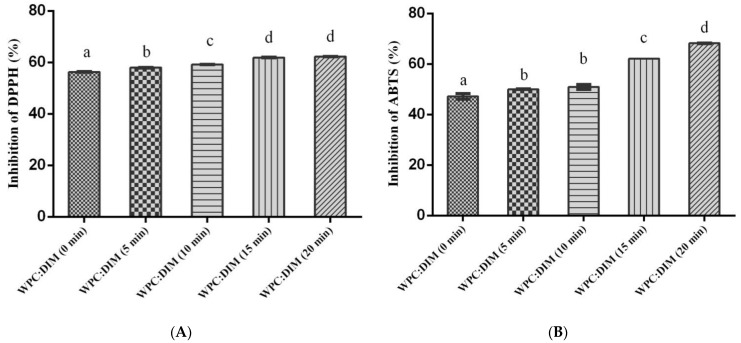
Effect of ultrasound treatment on antioxidant activity of WPC–DIM nanoparticles using two different methods (**A**) DPPH and (**B**) ABTS. Values with different letters a significant difference at (*p* < 0.05).

The antioxidant properties of WPC–DIM nanoparticles treated with various sonication times were determined using two different methods (DPPH and ABTS) and the results are shown in Figure 5. DPPH assay is an effective method to assess the antioxidant activity of many bioactive substances. DPPH is a purple stable radical solution at room temperature which changes to light yellow followed by the reduction by the addition of an antioxidant substance [39]. The scavenging effect of WPC–DIM nanoparticles with different ultrasound treatments on DPPH radicals demonstrated a sonication-time-dependent activity (Figure 5A). However, DPPH lacks selectivity due to spectral interferences. So, the use of the DPPH assay with another technique (ABTS) is recommended. As seen, WPC–DIM nanoparticles treated with various sonication times exhibited antioxidant activity because DIM has two N–H groups in its structure that could neutralize free radicals by donating H groups [40]. Our results revealed that the radical-scavenging activity of untreated DIM-loaded WPC nanoparticles (56%) significantly increased to almost 57, 59, 61, and 62% after ultrasound treatment for 5, 10, 15, and 20 min, respectively (*p* < 0.05) (Figure 5A). The ultrasound treatment of nanoparticles for 20 min showed the highest DPPH radical activity (62%). This could be explained by the fact that ultrasound treatment reduced particle size of the nanoparticles [19] and the nanometric size of the nanoparticles in the solution might facilitate a better contact surface area between the DPPH and H donator, providing the H atom access to the radical site [3]. Our results were supported by the previous literature, which reported a similar enhanced antioxidant activity by ultrasound treatment compared to untreated samples [30,39].

ABTS is a more versatile technique to determine the antiradical activity of substances in several media because it is not affected by the spectra of complex products. The solution has a blue/green color, but is decolorized by antioxidant compounds, and its characteristic absorption is decreased by a donating hydrogen [40]. In our study, ABTS results supported the findings of the DPPH assay. Figure 5B shows that the ABTS radical-scavenging activity of WPC–DIM nanoparticles increased after ultrasound treatment. The results show that untreated ultrasound nanoparticles’ ABTS radical activity was around 47%, which was significantly increased by ultrasound treatment with various sonication times (5, 10, 15, and 20 min), and maximum ABTS radical activity reached 68% upon 20 min of ultrasound treatment (*p* < 0.05) (Figure 5B). These results are consisted with previous findings on WPI–GA treated with different ultrasound times, who reported that the radical-scavenging activity of WPI–GA increased with sonication times [39].

## 4. Conclusions

In this work, WPC–DIM nanoparticles were prepared and treated with different sonication times (0–20 min). The effects of different sonication times on the nanoparticles were characterized by particles size, PDI, zeta potential, rheology, TEM, DSC, and FT-IR; antioxidant activity was also determined. Ultrasonication treatment for 5–20 min resulted in a significant decrease in the particle size from 265.95 ± 14.51 to 218.46 ± 9.37 nm, a reduction in the distribution, and an increase in the zeta potential value from −27.96 to −32 mV. The ultrasound treatment with different sonication times resulted in an improvement in encapsulation efficiency (EE%) as compared to the control sample. The ultrasound treatment had a significant effect on the apparent viscosity, and a decrease in the viscosity as a function of shear rate was observed with increasing sonication time. The transmission electronic microscopy (TEM) micrograph demonstrated that all of the formulations treated with different sonication times had a smooth and uniform spherical shape and that ultrasound treatment led to the reduction of particle size, especially after 20 min of ultrasonication. A significant impact was noted on the pH of the WPC–DIM nanoparticles after sonication due to ultrasound-induced changes in the protein structure. Ultrasound treatment affected the thermal properties of the nanoparticles and enhanced the denaturation peak T_d_ and the denaturation temperature range (ΔT_d_) in comparison to the untreated sample. The Fourier transform infrared (FT-IR) spectra demonstrated that ultrasound treatment had a remarkable effect on the secondary structure of the WPC–DIM nanoparticles, and that electrostatic interactions and hydrogen bonds between DIM and whey protein concentrate were strengthened. Moreover, DPPH scavenging activity and ABTS scavenging activity results confirmed the radical-scavenging activity of the WPC–DIM nanoparticles, and this was enhanced by ultrasonication.

It is concluded that physicochemical, rheological, microstructure, and thermal properties of WPC–DIM nanoparticles could be controlled and enhanced by varying the sonication time; therefore, it is considered an efficient method to develop DIM-loaded whey protein concentrate nanoparticles to be used for medical and nutritional applications.

## Figures and Tables

**Table 1:** Effect of ultrasound treatment on particle size, PDI, zeta potential, and encapsulation efficiency (EE%) of WPC–DIM nanoparticles.

Samples	Particle Size (nm)	PDI	Zeta Potential	EE%
WPC only	230.50 ± 11.66 ^ac^	0.41 ± 0.01 ^a^	−21.93 ± 0.55 ^a^	
WPC-DIM (0 min)	265.96 ± 14.5 ^b^	0.49 ± 0.02 ^b^	−27.96 ± 0.64 ^b^	76.46 ± 0.05 ^a^
WPC-DIM (5 min)	255.86 ± 2.55 ^ab^	0.48 ± 0.03 ^bc^	−30.26 ± 1.05 ^c^	77.36 ± 0.23 ^b^
WPC-DIM (10 min)	253.36 ± 12.46 ^ab^	0.45 ± 0.01 ^abc^	−30.40 ± 1.40 ^c^	79.03 ± 0.05 ^c^
WPC-DIM (15 min)	250.03 ± 16.87 ^abc^	0.43 ± 0.0 ^ac^	−30.76 ± 0.49 ^cd^	81.50 ± 0.10 ^d^
WPC-DIM (20 min)	218.46 ± 9.37 ^c^	0.43 ± 0.02 ^ac^	−32.83 ± 0.37 ^d^	88.51 ± 0.02 ^d^

Note: Values with different letters in the same column denote a significant difference at (*p* < 0.05).

**Table 2:** Effect of ultrasound treatment on rheological properties of WPC–DIM nanoparticles.

Samples	*η*_∞_ (mPa∙s) Infinite-Shear-Rate Viscosity	*k*_ᴑ_ Consistency Index	*n* Flow Index
WPC only	4.51 ± 0.27 ^ac^	3.55 ± 0.03 ^a^	−0.41 ± 0.02
WPC-DIM (0 min)	10.35 ± 0.14 ^b^	0.33 ± 0.04 ^b^	0.50 ± 0.03
WPC-DIM (5 min)	4.7 ± 0.24 ^ac^	1.01 ± 0.05 ^c^	−1.03 ± 0.02
WPC-DIM (10 min)	4.36 ± 0.36 ^acd^	1.02 ± 0.09 ^c^	−0.13 ± 0.02
WPC-DIM (15 min)	3.99 ± 0.19 ^cd^	0.11 ± 0.04 ^d^	0.23 ± 0.09
WPC-DIM (20 min)	2.34 ± 0.11 ^e^	0.27 ± 0.09 ^b^	0.12 ± 0.01

Note: Values with different letters in the same column denote a significant difference at (*p* < 0.05).

**Table 3:** Effect of ultrasound treatment on the color and pH of WPC–DIM nanoparticles.

Samples	Color Coordinates			pH
	L*	a*	b*	
WPC only	49.33 ± 3.63 ^a^	−1.92 ± 0.20 ^a^	15.36 ± 0.05 ^a^	
WPC-DIM (0 min)	64.80 ± 1.92 ^b^	−0.57 ± 0.07 ^bc^	19.28 ± 0.35 ^b^	6.95 ± 0.01 ^a^
WPC-DIM (5 min)	64.75 ± 2.32 ^b^	−0.53 ± 0.05 ^bc^	18.5 ± 0.60 ^b^	7.00 ± 0.01 ^b^
WPC-DIM (10 min)	64.99 ± 3.45 ^b^	−0.87 ± 0.09 ^cd^	19.27 ± 0.30 ^b^	7.01 ± 0.00 ^bc^
WPC-DIM (15 min)	64.78 ± 2.56 ^b^	−1.32 ± 0.31 ^d^	18.29 ± 0.22 ^b^	7.03 ± 0.01 ^c^
WPC-DIM (20 min)	66.87 ± 3.05 ^b^	−0.38 ± 0.11 ^b^	22.59 ± 0.39 ^c^	7.08 ± 0.00 ^d^

Note: Values with different letters in the same column denote a significant difference at (*p* < 0.05).

**Table 4:** Effect of ultrasound treatment on the onset temperature (T_on_), denaturation temperature range (ΔT_d_), peak denaturation temperature (T_d_), and denaturation enthalpy (ΔH_d_) of WPC–DIM nanoparticles.

Samples	T_on_ (°C)	ΔT_d_ (°C)	T_d_ (°C)	ΔH_d_ (mW/g)
WPC-DIM (0 min)	51	51–149	90.46	179.80
WPC-DIM (5 min)	54.8	54.8–151.58	90.57	173.70
WPC-DIM (10 min)	63.31	63.31–159.49	93.94	224.90
WPC-DIM (15 min)	61.15	61.15–158.02	94.96	165.40
WPC-DIM (20 min)	63.66	63.66–164.76	96.04	194.30

## Data Availability

Data is contained within the article.
